# A Catheter-Based Acoustic Interrogation Device for Monitoring Motility Dynamics of the Lower Esophageal Sphincter

**DOI:** 10.3390/s140814700

**Published:** 2014-08-12

**Authors:** Qian Lu, Orly Yadid-Pecht, Daniel C. Sadowski, Martin P. Mintchev

**Affiliations:** 1 Department of Electrical and Computer Engineering, University of Calgary, 2500 University Drive, N.W., Calgary, Alberta T2N1N4, Canada; E-Mails: qlu@ucalgary.ca (Q.L.); orly.yadid-pecht@ucalgary.ca (O.Y.-P.); 2 Faculty of Medicine, University of Alberta, 116 St, 85 Ave, Edmonton, Alberta T6G2R3, Canada; E-Mail: dan.sadowski@ualberta.ca

**Keywords:** lower esophageal sphincter (LES), oscillator, microphone, acoustic catheter

## Abstract

This paper presents novel minimally-invasive, catheter-based acoustic interrogation device for monitoring motility dynamics of the lower esophageal sphincter (LES). A micro-oscillator actively emitting sound wave at 16 kHz is located at one side of the LES, and a miniature microphone is located at the other side of the sphincter to capture the sound generated from the oscillator. Thus, the dynamics of the opening and closing of the LES can be quantitatively assessed. In this paper, experiments are conducted utilizing an LES motility dynamics simulator. The sound strength is captured by the microphone and is correlated to the level of LES opening and closing controlled by the simulator. Measurements from the simulator model show statistically significant (*p* < 0.05) Pearson correlation coefficients (0.905 on the average in quiet environment and 0.736 on the average in noisy environment, D.O.F. = 9). Measuring the level of LES opening and closing has the potential to become a valuable diagnostic technique for understanding LES dysfunction and the disorders associated with it.

## 1. Introduction

Lower esophageal sphincter (LES) is located between the esophagus and the stomach. The LES acts principally as a barrier against reflux of gastric content into the esophagus, while allowing antegrade passage of esophageal content into the stomach. Malfunction of the LES may cause gastroesophageal reflux disease (GERD), which is one of the most common gastrointestinal diseases. In 2004, approximately 20 percent of the United States population reported reflux symptoms that occurred at least weekly [1].

One of the primary malfunctions in LES is the phenomenon of transient inappropriate relaxations of the sphincter, resulting in permissive conditions for the content of the stomach to reflux into the esophagus resulting in various esophageal symptoms and mucosal damage. It has been hypothesized that such esophageal damage may be a precursor to esophageal cancer [2,3]. Existing methods for monitoring LES-related disorders include pH measurements, impedance monitoring, endoscopy, and esophageal manometry, as well as ultrasonic and sonic techniques [4–18]. However, none of these can directly assess the opening and the closing of the LES [19].

Esophageal pH monitoring can directly demonstrate the presence of acidic gastric content in the organ. Studies have shown that a pH value of less than 4 that has been continuously monitored for more than 5% of the test duration is diagnostic of GERD [4]. One of the primary causes of GERD is a malfunction of the lower esophageal sphincter (LES), which controls the flow of luminal content between the esophagus and the stomach. However, pH monitoring cannot detect non-acidic GER episodes, which is often experienced by patients taking acid-suppressive medication [10]. Hence, multichannel intraluminal impedance (MII) measurements have been integrated with pH monitoring to increase system sensitivity in detecting both acidic and non-acidic GER episodes [11]. However, even the combined MII-pH technique is still considered an indirect measurement of LES opening and closing. As a result, the degree of LES opening and closing and its corresponding temporal dynamics remains inaccessible, particularly in ambulatory conditions [19].

Endoscopic investigation is an alternative approach for LES monitoring. An endoscope, which is a thin and long tube with a camera at the proximal end, is introduced through the mouth and the esophagus to observe the mucosal layer of the gastrointestinal tract for a short period of time. The traditional stationary endoscopic approach mainly investigates morphological changes in the LES region [5]. If needed, biopsy can also be performed to identify histological lesions [6], but long-term, ambulatory studies using this technique are impossible. Even miniature endoscopic catheters cannot be reliably utilized for a long-term, ambulatory monitoring of the motility dynamics of the LES due to numerous limitations, which include the effects of swallowing, eating, and esophageal wall collapsing over the catheter at rest [20].

Esophageal manometric abnormalities have been utilized in the investigation of esophageal function since 1970s when technology was developed to allow for a proper recording of esophageal pressure dynamics [7]. During esophageal manometry testing, a thin, pressure-sensitive tube is transnasally or transorally inserted into the stomach. Once in place, the tube is pulled slowly back into the esophagus. The patient is asked to swallow and the change of pressure during swallowing is recorded and stored into a computer for data analysis. The purpose of esophageal manometry is to assess the peristaltic activity of the proximal and distal esophagus. Usually, basal LES pressures less than 15 mmHg are normal, and the duration of LES relaxation is usually less than 9 s [8,9]. Manometry can indirectly monitor the opening and closing of the LES. However, still, there are numerous limitations. A recent review article [19] clearly states that even the combined monitoring of intraluminal pressure, pH and impedance cannot fill the void of a lacking technique to monitor reliably and directly the movement and location of the gastroesophageal junction over a prolonged period of time.

Intraluminal ultrasonic techniques are powerful tools to study esophageal motility function and dysfunction in vivo in humans [12,13]. Ultrasonic images can assess the cross-sectional structure of the esophagus and can depict important physiological information. For example, the cross-sectional area (CSA) of the esophageal lumen during liquid swallows and liquid gastroesophageal reflux can be measured by a probe carrying a circumferential array of miniature ultrasonic sensors on a single site of an intraluminal catheter to inspect visually the cross-sectional images, the results showing that the esophageal CSAs during GER are significantly greater in GER patients [14]. However, this cannot be done directly at the level of the LES itself, because it would interfere with its function. Analysis of esophageal content and dimensions during transient LES relaxations can be performed as well [15–17], but, again, without directly recording the opening and the closing of the sphincter.

Sonic signals generated from stomach during peristalsis could be clinically useful for the assessment of the LES function [18,21]. For example, several piezoelectric transducers were placed externally either on the sternum or on the back of the patient to capture the infrasound signals associated with vibrations originating at the LES area [18]. However, the sensitivity and the accuracy of this acoustic system for monitoring gastro-esophageal motility dysfunctions suffer from the weak sub-audible signals that are usually contaminated with noises generated from other internal organs or by the external environment.

All of the above-mentioned techniques exhibit respective drawbacks related to low accuracy and sensitivity. In addition, these methods are not able to provide quantitative analysis of the level of the opening or the closing of the LES. A new method that can directly and quantitatively measure the level of the opening and the closing of the LES in a minimally invasive fashion, and at the same time provide long-term (24–48 h), easily readable data, is greatly needed [19]. Interestingly, such technique would be applicable not only in GERD monitoring, but probably also in assessing other esophageal motility disorders, including achalasia, gastroesophageal junction obstruction, esophageal spasm, nutcracker esophagus and hypertensive lower esophageal sphincter [22].

## 2. Proposed Method

### 2.1. The Hypothesis

**Figure 1.**
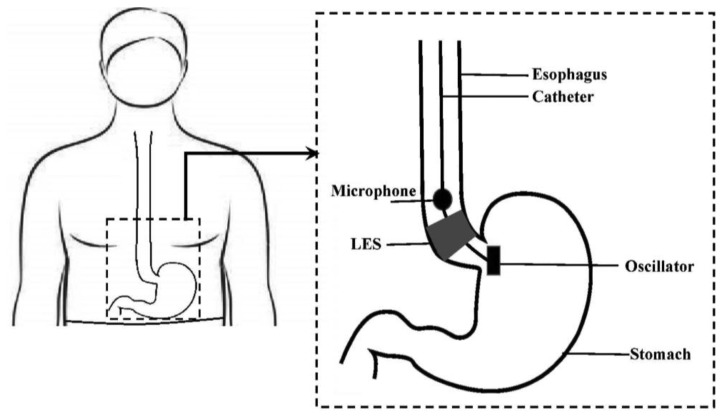
Concept of the proposed method.

As the LES is closing or opening, a microphone above the LES would gradually detect lower-power signal from a sound-generating, fixed-frequency oscillator placed below the LES, and the area of LES opening would be highly correlated with the detected acoustic signal amplitude at that fixed frequency. Figure 1 shows the concept of the proposed method.

### 2.2. Implementation

**Figure 2.**
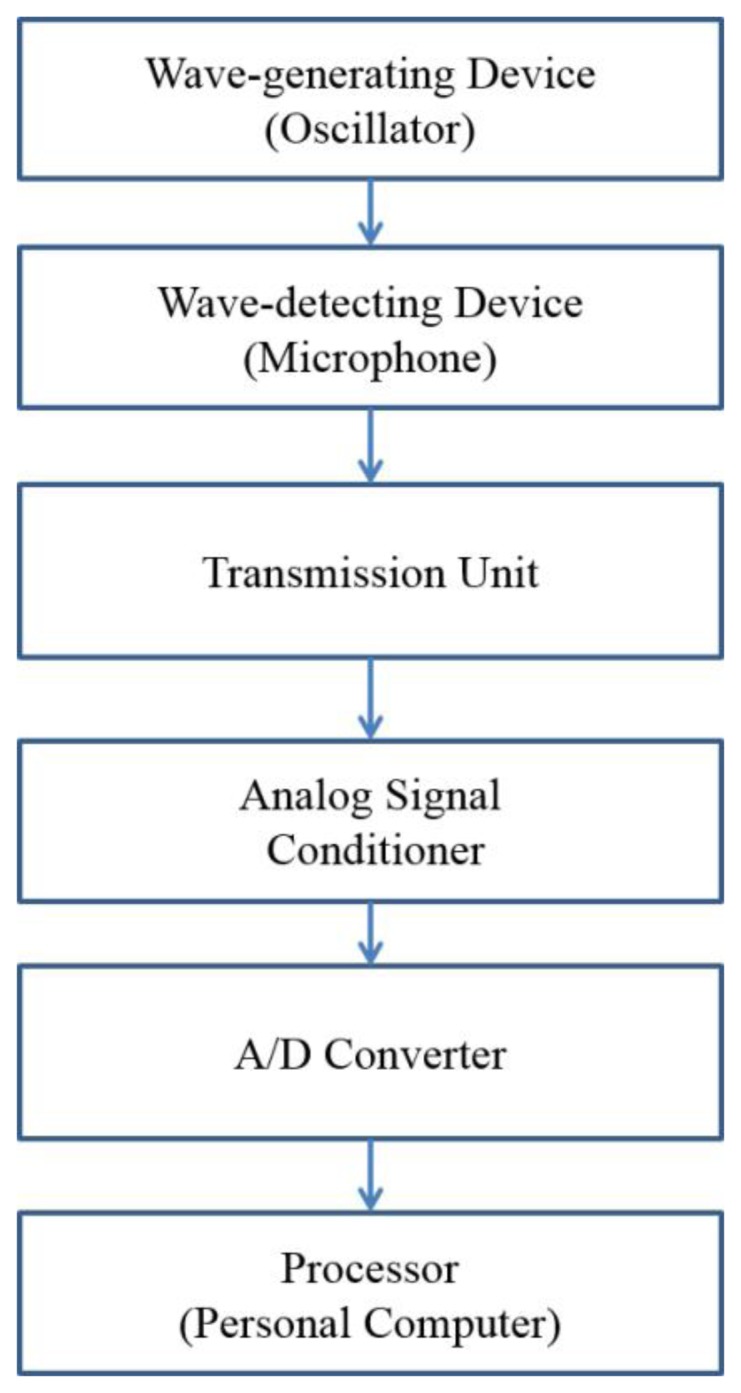
Block diagram of the proposed system.

The proposed method can be implemented on a catheter-based device comprising: (1) oscillator-microphone pair; (2) data transmission unit and (3) data processing unit. As depicted in Figure 1, the sound signals are transmitted in real-time from the microphone via wireless transmission unit and processed by a data processing unit which further includes an analog signal conditioner, analog-to-digital (A/D) converter and a personal computer. Figure 2 shows the block diagram of the proposed system.

#### 2.2.1. Catheter Design

**Figure 3.**
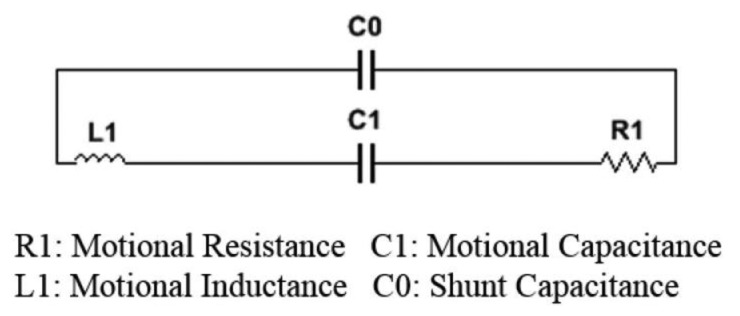
Equivalent circuit of the CX-2V quartz crystal.

The wave-generating device is implemented as an acoustic oscillator using CX-2V (STATEK, Orange, CA, USA) quartz crystal integrated circuit, routinely utilized to generate a signal in the frequency range from 16 to 600 kHz. Figure 3 shows the equivalent circuit of the CX-2V quartz crystal. The wave-detecting device is the 2.5-mm diameter B6W4 microphone (Countryman Associates, Menlo Park, CA, USA), which can provide high signal-to-noise ratio, high sensitivity and good noise-shrouding capability. The catheter is comprised by one oscillator and one microphone and is covered by silicone tubing for water and acid resistivity. The diameter of the catheter is 0.33 cm. The LES in humans is recognized as a zone of high pressure, 2 to 4 cm in length [23]. The size of manometric pressure probes used in clinical practice ranges from 0.3–0.45 cm in diameter, which is considered to have minor influence on LES pressure measurements [24]. Therefore, the impact of the proposed catheter on the LES and its vicinity should be minimal and comparable to that of a clinical manometry study.

#### 2.2.2. Data Transmission

In our system, the data transmission unit is a wireless transmitter-receiver pair. The pair includes one analog transmitter (UR1, Shure, Niles, IL, USA) and one analog receiver (UR4D, Shure, Niles, IL, USA). The transmitter transmits in real time the analog signals captured by the microphone to the outside receiver.

#### 2.2.3. Data Processing

The embedded analog signal conditioner with UR4D receiver conditions the analog signals from the transmission unit and sends them for digitization to an A/D converter (NI-9234, National Instruments, Austin, TX, USA) with a sampling rate of 51.2 kHz. The digitized signals are then processed by a processor (personal computer) and the NI Sound and Vibration Toolkit software package (National Instruments, Austin, TX, USA), which can display in real-time the information related to the wave signals detected by the microphone.

## 3. Experimental Verification

### 3.1. LES Motility Dynamics Simulator Model

**Figure 4.**
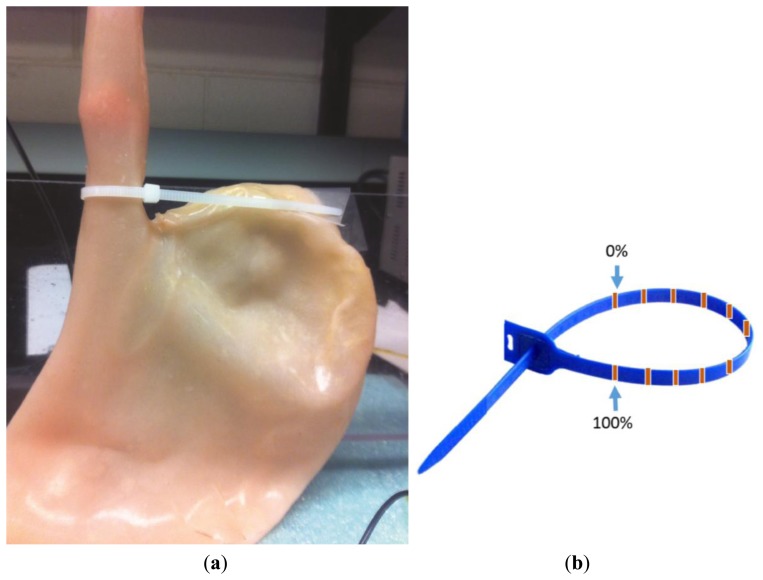
(**a**) Stomach model; (**b**) Plastic seal.

In order to test the device in-vitro, a LES dynamic simulator model has been developed. The model included a silicone stomach model (Simulab Corporation, Seattle, WA, USA) having a model esophagus (Figure 4a). The silicone stomach was vertically immobilized by a mechanical holder. For testing, the catheter was inserted into the experimental model to monitor shape changes. A plastic seal with labeling was employed to control the percentage of the LES opening (Figure 4b). The LES was totally open when the seal was at the position labeled “0%” and was totally closed when the seal was moved to the position labeled “100%”. The distance between the two labels was equally divided to indicate different levels of opening or closing of the LES.

The purpose of the simulator was to evaluate the performance of the acoustic catheter and to determine its suitability for in-vivo testing in a real esophagus. The stomach model was filled with physiological saline to simulate gastric content. We considered three different scenarios: empty, half-full and full stomach model. Among them, we observed that the biggest signal attenuation occurred at a full stomach. Therefore, we conducted experiments utilizing the full stomach model. Using this model, the obtained measurements from the acoustic system were correlated to the percentage of simulated LES opening.

### 3.2. Method

In order to study the relationship between the degree of the LES opening and the strength of the sound recorded by the microphone, eleven groups of data were collected in a quiet environment and another eleven groups of data were collected in a noisy environment using the LES motility dynamics simulator model. Eleven different percentages were introduced, “0”, meaning LES was totally open, “10”, meaning LES was 10% closed, “20”, meaning LES is 20% closed, and so on. “100” indicated that the LES was totally closed.

During the first two trials, the data were collected in a quiet environment. To test the robustness of this method, in the third and fourth trial, the same test incorporating the same procedures was performed, with an addition of random noise during the recording. The noise came from purposely stirring a bottle of water directly near the crystal in order to mimic the sounds from gastric juice movements in the stomach. Before the experiments, the oscillator circuit was turned on and the distance between the oscillator and the microphone was fixed at 5 cm. For each recording, the sound signal was recorded for 30 s. Then, the plastic seal was tightened to the next level to close the LES further, and the recording was repeated.

### 3.3. Data Analysis

The sound wave strength at different LES openings was calculated by averaging the signals within each 30-seconds time interval. Pearson's correlation coefficients were then calculated to study the relationship between the signal strength and the level of LES opening or closing [25]:

(1) $$r=\frac{\sum_{i=1}^{n}(X_{i}-\bar{X})(Y_{i}-\bar{Y})}{\sqrt{\sum_{i=1}^{n}{\left(X_{i}-\bar{X}\right)}^{2}}\sqrt{\sum_{i=1}^{n}{\left(Y_{i}-\bar{Y}\right)}^{2}}}$$

where *r* is the Pearson correlation coefficient, *X* is one of the monitored variables, and *Y* is the other. Positive values of *r* indicate a tendency of *X* and *Y* to increase together. When *r* is negative, large values of *X* are associated with small values of *Y*.

## 4. Results and Discussion

### 4.1. Experimental Results

**Figure 5.**
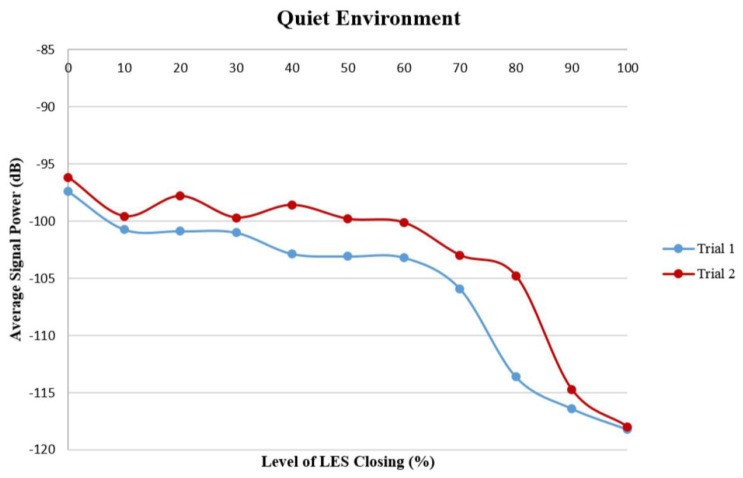
Two different tests of Average Signal Power *vs*. Level of lower esophageal sphincter (LES) Closing (in %) in a quiet environment.

Figure 5 shows two sets of data acquired during different experiments, representing average sound wave strength values at eleven levels of LES opening captured by our device in a quiet environment. A decreasing trend is clearly evident. The sound wave strength detected by the microphone decreased approximately by an order of 2 when the level of LES opening changed from 100% to 0%.

**Figure 6.**
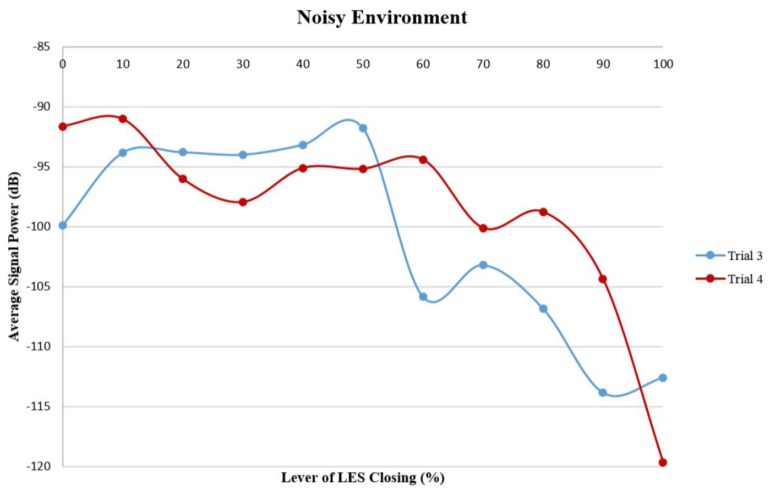
Two different tests of Average Signal Power *vs*. Level of LES Closing (in %) in a noisy environment.

Figure 6 shows another two sets of data acquired at different experiments, representing the average sound wave strength values at eleven levels of LES opening in a noisy environment, which depicts the same decreasing trend by similar order of magnitude.

Table 1 shows the Pearson correlation coefficients of the level of LES opening and the signal strength at 16 kHz frequency in both quiet and noisy environments. The Pearson correlation coefficients in all data sets were higher than the value for the chosen level of significance (*p* < 0.05), which indicated that the change in the strength of the sound signals at 16 kHz frequency was significantly correlated with the level of LES opening or closing.

### 4.2. Discussion

Present techniques for diagnosing GERD focus on change of parameters, such as pH, impedance or pressure caused by the movement of gastric contents during a reflux event. To our knowledge, the present study is the first attempt to directly monitor the levels of LES opening or closing. The designed sensor system explicitly demonstrated that sound strength is a very effective parameter for detecting the level of LES opening or closing. Direct monitoring of LES opening makes it possible to study other LES-related diseases, for example, achalasia which is a failure of the LES to relax.

Compared to eventual thin endoscopic image-based monitoring, the acoustic catheter-based design not only allows ambulatory monitoring for over 24 h, but also provides fast and informative digital signal processing to indicate the exact dynamics of the LES without an extensive image reading. The quantitative result of such ambulatory monitoring can facilitate physicians to better understand LES-related diseases.

Our goal was to develop a thin acoustic catheter system capable of prolonged ambulatory recording (24–48 h) of the LES motility dynamics, which would have the potential to take the assessment of the LES function to a new level, including monitoring of transient lower esophageal sphincter relaxations (tLESRs). Further testing on chronic animal models and human volunteers is needed to determine whether this goal can be achieved.

Noises from physiological activity and the surrounding environment can potentially affect the performance of the proposed acoustic device. Noise-removal filters can be investigated in order to improve performance. A two-microphone design (including a second microphone positioned in mid-esophagus on the same catheter) should be able to identify motion artifacts, swallowing, eating and drinking, but not LES motility. An adaptive filtering algorithm can then be utilized to eliminate such non-LES related events in real time [26].

## 5. Conclusion

A miniature, longitudinal, catheter-based acoustic oscillator system has been implemented for the detection and quantification of LES opening levels. The system was tested on a custom-designed LES simulator and a series of preliminary experiments have been performed to quantify the relationship between the signal strength at 16 kHz and the levels of LES opening. It has been clearly demonstrated that the more the LES closed, the lower the sound strength detected by the microphone became. Furthermore, according to statistical Pearson correlation coefficient analysis, a statistically significant positive correlation was attained, regardless of the presence of external noises.

## Figures and Tables

**Table 1.** Average Pearson correlation coefficients of the level of LES opening and the sound strength in quiet and noisy environments. The borderline statistical significance level (*p* = 0.05) is also included.

Degrees of Freedom = 9	In Quiet Environment	In Noisy Environment	*p* = 0.05 Level
Pearson correlation coefficient r	r = 0.886 (Trial 1)	r = 0.792 (Trial 3)	0.602

	r = 0.915 (Trial 2)	r = 0.680 (Trial 4)	0.602
